# Bone Marrow Is a Major Parasite Reservoir in Plasmodium vivax Infection

**DOI:** 10.1128/mBio.00625-18

**Published:** 2018-05-08

**Authors:** Nicanor Obaldia, Elamaran Meibalan, Juliana M. Sa, Siyuan Ma, Martha A. Clark, Pedro Mejia, Roberto R. Moraes Barros, William Otero, Marcelo U. Ferreira, James R. Mitchell, Danny A. Milner, Curtis Huttenhower, Dyann F. Wirth, Manoj T. Duraisingh, Thomas E. Wellems, Matthias Marti

**Affiliations:** a Department of Immunology and Infectious Diseases, Harvard School of Public Health, Boston, Massachusetts, USA; b Tropical Medicine Research, Panama City, Panama; c Instituto Conmemorativo Gorgas de Estudios de la Salud, Panama City, Panama; d Center for Excellence in Vascular Biology, Brigham and Women’s Hospital, Boston, Massachusetts, USA; e Laboratory of Malaria and Vector Research, National Institute of Allergy and Infectious Diseases, Bethesda, Maryland, USA; f Department of Biostatistics, Harvard School of Public Health, Boston, Massachusetts, USA; g Department of Parasitology, Institute of Biomedical Sciences, University of Sao Paulo, Sao Paulo, SP, Brazil; h Department of Genetics and Complex Diseases, Harvard School of Public Health, Boston, Massachusetts, USA; i Wellcome Centre for Molecular Parasitology, University of Glasgow, Glasgow, United Kingdom; University of Pittsburgh

**Keywords:** *Aotus*, real-time PCR, *Saimiri*, blood-stage parasites, gametocytes, immunohistochemistry, laboratory animal models, malaria, transcriptome

## Abstract

Plasmodium vivax causes heavy burdens of disease across malarious regions worldwide. Mature P. vivax asexual and transmissive gametocyte stages occur in the blood circulation, and it is often assumed that accumulation/sequestration in tissues is not an important phase in their development. Here, we present a systematic study of P. vivax stage distributions in infected tissues of nonhuman primate (NHP) malaria models as well as in blood from human infections. In a comparative analysis of the transcriptomes of P. vivax and Plasmodium falciparum blood-stage parasites, we found a conserved cascade of stage-specific gene expression despite the greatly different gametocyte maturity times of these two species. Using this knowledge, we validated a set of conserved asexual- and gametocyte-stage markers both by quantitative real-time PCR and by antibody assays of peripheral blood samples from infected patients and NHP (*Aotus* sp.). Histological analyses of P. vivax parasites in organs of 13 infected NHP (*Aotus* and *Saimiri* species) demonstrated a major fraction of immature gametocytes in the parenchyma of the bone marrow, while asexual schizont forms were enriched to a somewhat lesser extent in this region of the bone marrow as well as in sinusoids of the liver. These findings suggest that the bone marrow is an important reservoir for gametocyte development and proliferation of malaria parasites.

## IMPORTANCE

Plasmodium vivax malaria continues to cause major public health burdens worldwide. Yet, significant knowledge gaps in the basic biology and epidemiology of P. vivax malaria remain, largely due to limited available tools for research and diagnostics. Here, we present a systematic examination of tissue sequestration during P. vivax infection. Studies of nonhuman primates and malaria patients revealed enrichment of developing sexual stages (gametocytes) and mature replicative stages (schizonts) in the bone marrow and liver, relative to those present in peripheral blood. Identification of the bone marrow as a major P. vivax tissue reservoir has important implications for parasite diagnosis and treatment.

## INTRODUCTION

Nearly half of the world’s population lives in areas at risk of malaria transmission (1). In regions outside of sub-Saharan Africa where malaria is endemic, Plasmodium vivax dominates the public health burden from this infectious disease (2–4). P. vivax is also increasingly recognized in Africa, where it has been demonstrated to cause infections of Duffy-negative individuals (5). First-line treatment with chloroquine frequently fails against drug-resistant P. vivax in the Indonesian Archipelago, while the use of primaquine is dangerous to patients with glucose-6-phosphate dehydrogenase deficiency and can be unreliable against the dormant liver-stage forms (hypnozoites) of some strains (6, 7). The human burden of P. vivax malaria is gaining greater attention, notably in areas where it persists after the elimination of P. falciparum by malaria control programs (8). Yet, research on P. vivax has generally lagged behind research on P. falciparum (3, 9, 10), while much of our knowledge of P. vivax biology has been founded on experimental infections of nonhuman primates (NHP) and mosquitoes (11). Practical methods for continuous *in vitro* culture of P. vivax remain elusive (12–16), and genetic modification of the parasite has been possible only with the use of NHP models (17). Malaria control and elimination agendas have highlighted the need for research advances as an important foundation for progress against P. vivax (18, 19).

Transmission of *Plasmodium* parasites depends upon the presence of male and female gametocytes in blood ingested by feeding mosquitoes. P. vivax gametocytes mature much more rapidly than those of P. falciparum and can be observed in the bloodstream 3 to 5 days after the first microscopic detection of asexually replicating parasites, a finding that helps to explain why transmission can happen before or at the first development of vivax malaria symptoms (3, 9, 10, 20, 21). While fundamental features of P. vivax and P. falciparum transmission biology are likely shared, P. falciparum gametocytes have conspicuously distinct morphologies, require 8 to 10 days to mature, and accumulate/sequester during their development in the bone marrow parenchyma before emergence into the blood circulation (22, 23). P. vivax invades reticulocytes that are prevalent in the bone marrow parenchyma (24), and proportionally increased distributions of gametocytes and young parasite stages have been detected in the marrow aspirates of a clinical case (25). Together, these observations suggest a role for the bone marrow in P. vivax infection. Here, we present a systematic investigation of P. vivax tissue distributions and provide further evidence for enrichment of transmission and replicative stages in the bone marrow and liver, relative to peripheral blood.

## RESULTS

### Shared patterns of transcription in the development of P. vivax and P. falciparum gametocytes.

P. vivax orthologs of conserved P. falciparum gametocyte and gamete antigens (and transmission-blocking vaccine candidates), e.g., Pvs25, Pvs28, Pvs48/45, and Pvs230, provide markers that can complement morphological observations and likewise have diagnostic and vaccine potential (26–28). To characterize the expression profiles of P. vivax gametocytes and search further for stage-specific markers, we analyzed and compared the transcriptomes of P. vivax and P. falciparum gametocytes over the course of their development to maturity.

Our previous work provided a comprehensive analysis of temporal relationships among P. falciparum gametocyte transcripts, including the transcripts of 591 genes that grouped into 29 clusters (established by ≥5 coexpressed genes), from the onset of gametocytogenesis to final maturation (29). These previous results identified (i) 5 transcript clusters from gametocyte ring (GR) stages during the first day of development, (ii) 15 transcript clusters from immature gametocyte (IG) stages during days 2 to 6 of development, and (iii) 9 transcript clusters from P. falciparum mature gametocytes (MG) in their final 2 days of development and maturation. To develop a corresponding analysis of P. vivax transcript patterns, we identified a subset of P. falciparum gametocyte-expressed genes having syntenic orthologs in P. vivax. Of this subset’s 591 P. falciparum genes in 29 clusters, 527 showed evidence of a syntenic ortholog in P. vivax, whereas 64 did not have any clear ortholog and were therefore classified as missing from P. vivax (see Table S1 in the supplemental material). Among the P. falciparum gametocyte transcripts missing from P. vivax, many encode known P. falciparum gametocyte-exported proteins (GEXP) (30).

10.1128/mBio.00625-18.5 TABLE S1  Syntenic orthologs between P. falciparum and P. vivax, classified by stage-specific annotations for P. falciparum (29). Download TABLE S1, XLSX file, 0.2 MB. Copyright © 2018 Obaldia et al. 2018 Obaldia et al. This content is distributed under the terms of the Creative Commons Attribution 4.0 International license.

**FIG 1:**
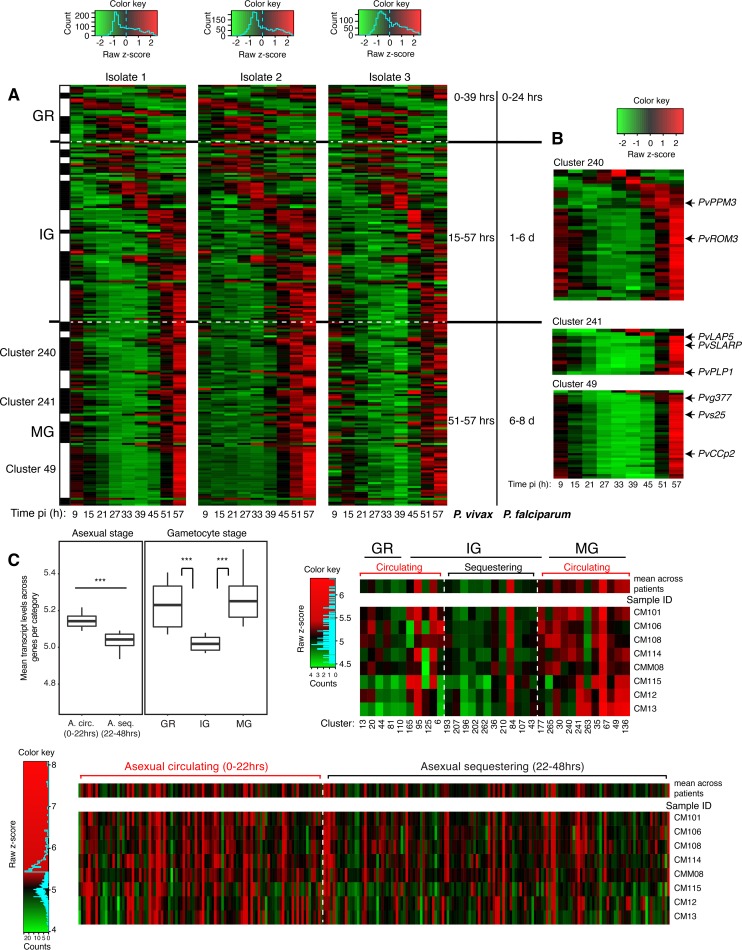
Stage-specific P. vivax gene expression *ex vivo* and in patients. (A) P. vivax gametocyte transcriptional dynamics *ex vivo*. P. vivax *ex vivo* transcriptome data from three patient isolates (31) were reanalyzed for gametocyte transcriptional dynamics. In the heat map, P. vivax orthologs from P. falciparum gametocyte genes were sorted along the developmental cycle according to stage specificity of previously defined clusters of P. falciparum coexpression analysis (29) (see Materials and Methods). The progression of transcript orthologs through the stages of P. vivax and P. falciparum gametocyte development was similar, although the period of the immature gametocyte cycle was greatly extended in P. falciparum compared to P. vivax. (B) Three selected transcriptional clusters including P. vivax markers *PvPPM3* (cluster 240), *PvLAP5* (cluster 241), and *Pvg377* and *Pvs25* (both cluster 49). (C) P. vivax gene expression in patient samples. P. vivax transcriptome data from 8 patient isolates (CM12, CM13, CM101, CM106, CM108, CM114, CM115, and CMM08) (34) were reanalyzed to define gametocyte transcript abundance in patient peripheral blood. (Left) Box plot showing mean expression across all genes representing one of five categories: A. circ, asexual 0 to 22 hpi; A. seq., 22 to 48 hpi (gametocyte GR, IG, and MG). ***, *P* < 0.005, paired *t* test. (Right) Heat map showing mean expression per cluster (sorted as for panel A) for GR, IG, and MG stages. (Bottom) Heat map showing mean expression of young circulating asexual stages and more mature asexual stages which accumulate/sequester in tissues. Dashed lines demarcate categories defined previously for P. falciparum (29).

In concert with comparative analysis of gametocyte transcripts, we analyzed the orthologs of P. falciparum genes previously shown to be expressed in P. vivax asexual blood stages (31). In experiments with three patient isolates, results from 9- to 57-h periods of *ex vivo* P. vivax culture revealed cascades of coexpressed genes in similar patterns (Fig. S1A) (both P. falciparum and P. vivax have 48-h asexual stage cycles). Consistent with previous findings (31), transcripts from ring stages were readily detected at the 51- and 57-h time points, indicating successful reinvasion of merozoites after an initial cycle of asexual parasite development. Using the P. vivax orthologs of P. falciparum gametocyte-expressed genes (Table S1), we analyzed the data for expression clusters of P. vivax sexual stages on the 9- to 57-h timeline. Progression through GR, IG, and MG clusters was evident, with strongest signals from the MG clusters at the 57-h time point (Fig. 1A and B). This time to the appearance of MG clusters in P. vivax *ex vivo* samples is consistent with microscopic observations of gametocyte development within 3 days (32) and contrasts with the much longer periods reported for the development of mature P. falciparum gametocytes (5 to 7 days for evidence of MG transcripts from *in vivo* samples; 13 days or more for P. falciparum MG to be infectious to mosquitoes) (29, 33). Interestingly, a subset of putative IG clusters in P. vivax was not observed until the 51- to 57-h *ex vivo* time points and therefore was grouped with the MG clusters. Taken together, these data support the presence of evolutionarily conserved cascades of transcriptional clusters in P. vivax and P. falciparum gametocyte development, although these two *Plasmodium* species exhibit very different maturity times and morphologies of their gametocytes.

10.1128/mBio.00625-18.1 FIG S1  Asexual gene expression across the P. vivax *ex vivo* cycle and schematic of gametocyte marker selection process. (A) P. vivax *ex vivo* transcriptome data from three patient isolates (31) were reanalyzed to characterize and display the asexual-stage transcriptional dynamics. (B) Schematic of the selection process for stage-specific gametocyte marker candidates, including stage specificity in P. vivax transcriptional data, evidence of protein expression by mass spectrometry, no male or female sex specificity in a P. falciparum ortholog, and presence of introns. Download FIG S1, TIF file, 4.9 MB. Copyright © 2018 Obaldia et al. 2018 Obaldia et al. This content is distributed under the terms of the Creative Commons Attribution 4.0 International license.

### Relative underrepresentation of P. vivax immature gametocyte and late asexual stages in patient blood.

Our previous study showed that the stage-specific accumulation/sequestration of P. falciparum parasites could be inferred from the transcription profiles of blood samples (29). To develop a corresponding analysis of P. vivax stages, we examined the transcription data from a previously published investigation of samples from eight P. vivax-infected patients (34). Orthologs of the gametocyte- and asexual-specific transcripts were assigned to P. vivax clusters as described above and grouped as defined for the P. falciparum study (29). In analysis of the sexual-stage clusters, we found that transcription levels in the IG clusters were lower than in GR clusters (paired *t* test *P* = 0.0028) or MG clusters (paired *t* test *P* = 0.0025) (Fig. 1C). Comparison of transcript levels in the asexual clusters revealed a significant underrepresentation of signal from late asexual (>22 h postinvasion [hpi]) relative to early asexual stages of P. vivax (<22 hpi) (paired *t* test *P* = 0.0015) (Fig. 1C). This finding from the asexual stages is consistent with conclusions that ring-stage parasites are present at greater prevalence than trophozoites and schizonts in the blood of P. vivax*-*infected patients, and these more mature stages of P. vivax accumulate/sequester (35, 36). Further, results from our transcriptome-cluster analysis of GR, IG, and MG provided quantitative evidence for differential representation of IG stages in circulating blood, which may be attributed to their accumulation/sequestration in other tissues.

### Selection of stage-specific markers and validation by quantitative real-time PCR.

Stage-specific quantitative real-time PCR (qRT-PCR) markers to investigate tissue accumulation/sequestration were tested and validated with blood samples from Aotus lemurinus lemurinus monkeys (37, 38) infected with P. vivax strain AMRU-I or SAL-I (39, 40) (Fig. S2 and S3; Table S2). Markers for this purpose were required to (i) be specific for P. vivax gametocyte or asexual stages, (ii) have orthology to a P. falciparum marker, and (iii) span an intron splice site, to avoid artifacts from spurious amplifications of a genomic DNA (gDNA) template (Fig. S1B). In an approach analogous to our previous identification of P. falciparum stage-specific markers for use in multiplexed qRT-PCRs (41, 42), we used *ex vivo* and *in vivo* transcript data plus available proteomics information (http://www.PlasmoDB.org) to develop a short list of 16 P. vivax candidates (Table S3). We also included *PVX18s rRNA* as a highly expressed constitutive transcript for qRT-PCR assay normalization and the *Pvs25* sequence as a reference standard for detection of mature gametocytes (27, 28). Optimization of the exon-spanning primer sets was performed using synthetic DNAs representing the expected cDNAs from each mature mRNA sequence lacking the intron. We tested for amplification efficiency in dilution series of these synthetic cDNA templates, and 10 primer pairs showed PCR amplification efficiency of >90% (Table S3). We trimmed the candidate list further by confirming there was no spurious amplification from the P. vivax gDNA template. The above selection criteria were met by primer sets for two candidates: (i) *PVX_117730*, encoding putative protein phosphatase PvPPM3, and (ii) *PVX_117900*, encoding the P. vivax gamete surface antigen PvLAP5 (43), an ortholog of PfFNPA (PF3D7_1451600; PF14_0491) in P. falciparum (44). The *Pvs25* and *PVX18s rRNA* primer pairs were also checked for efficient product amplification, but with P. vivax gDNA, as these genes lack introns and were shown not to yield products with cDNA or gDNA from uninfected *Aotus* NHP or humans. Identities of the *PVX_117730*, *PVX_117900*, *Pvs25*, and *PVX18s rRNA* amplicons were verified by agarose gel electrophoresis and Sanger sequencing (Fig. S4).

10.1128/mBio.00625-18.2 FIG S2  Experimental timelines from studies of P. vivax-infected nonhuman primates. (A) Schematic of inoculation and blood sample collection procedures. Four *Aotus* monkeys each were inoculated with P. vivax AMRU-I and SAL-I samples from infected donor animals, and parasite population expansions were monitored by microscopic analysis of blood smears. At peak parasitemia (P. vivax AMRU-I = 47.9 ± 16.9; P. vivax SAL-I = 61.8 ± 23.9 [mean peak parasitemia ± standard deviation, × 10^3^ parasites per µl] [Table S2]), a maximum volume of 3.5 ml of blood was collected for assays and analysis. (B) Plots of parasitemia in infected *Aotus* animals and corresponding *ex vivo* cultures. (Top) Parasitemia curves from P. vivax SAL-I-infected *Aotus* monkeys (left) and corresponding *ex vivo* cultures (right). (Bottom) Parasitemia curves from P. vivax AMRU-I-infected *Aotus* monkeys (left) and corresponding *ex vivo* cultures (right). Download FIG S2, TIF file, 4.5 MB. Copyright © 2018 Obaldia et al. 2018 Obaldia et al. This content is distributed under the terms of the Creative Commons Attribution 4.0 International license.

10.1128/mBio.00625-18.3 FIG S3  Photograph of a Percoll band carrying P. vivax*-*infected red blood cells and images of isolated parasite stages. (A) Images of the Percoll gradient layer and recovered P. vivax-infected *Aotus* RBCs. Giemsa-stained cells show that the interface band was highly enriched in late asexual and gametocyte stages, while the pellet was enriched in ring-stage parasites. (B) Images of P. vivax-infected *Aotus* RBCs after a 48-h *ex vivo* culture (AMRU-I). Mature as well as immature asexual blood stages were evident, including gametocytes. Download FIG S3, TIF file, 32.7 MB. Copyright © 2018 Obaldia et al. 2018 Obaldia et al. This content is distributed under the terms of the Creative Commons Attribution 4.0 International license.

10.1128/mBio.00625-18.4 FIG S4  qRT-PCR optimization for transcript detection and determinations of parasite burdens. (A) PCR products from amplification of P. vivax cDNA. RNA was extracted from *Aotus* blood infected with P. vivax AMRU-I. Bands of expected sizes are evident for *PVX_117900 (PvLAP5)* (lane 2), *PVX_117730 (PvPPM3)* (lane 3), and *Pvs25* (lane 4). (B) Amplification from P. vivax gDNA. *Pvs25* shows efficient amplification. There was no signal over the no-template control (NTC) for *PVX_117900*, *PVX_117730*, or *PVX_091645* when we used exon-spanning primer pairs. (C) Parasite burdens in blood, determined by qRT-PCR versus microscopy. (Left) Parasitemia based on counts from Giemsa-stained blood smears correlated with *PVX18s rRNA* levels determined by qRT-PCR of AMRU-I cDNA (samples from 13 animals, represented by dots). (Right) Gametocytemia based on microscopy counts from Giemsa-stained blood smears correlated with *PvLAP5* (black) and *Pvs25* (red) from the same *Aotus* blood samples. qRT-PCR data were normalized by amount of input RNA used per reaction mixture. Linear regression analysis demonstrated a highly significant correlation between the two measurements. (D) Parasite burden in blood versus liver and bone marrow. Parasite burdens based on pLDH counts from Fig. 3B in bone marrow and liver were correlated with *PVX18s rRNA* transcript levels in peripheral blood from the same monkey (dots represent results from 13 monkeys). For all correlations, Spearman *R* (*r*^2^) and *P* values were computed using a nonparametric Spearman correlation. Download FIG S4, TIF file, 7.1 MB. Copyright © 2018 Obaldia et al. 2018 Obaldia et al. This content is distributed under the terms of the Creative Commons Attribution 4.0 International license.

10.1128/mBio.00625-18.6 TABLE S2  Details of *Aotus* infections with P. vivax. Download TABLE S2, XLSX file, 0.01 MB. Copyright © 2018 Obaldia et al. 2018 Obaldia et al. This content is distributed under the terms of the Creative Commons Attribution 4.0 International license.

10.1128/mBio.00625-18.7 TABLE S3  Evaluation of stage-specific markers. Download TABLE S3, XLSX file, 0.01 MB. Copyright © 2018 Obaldia et al. 2018 Obaldia et al. This content is distributed under the terms of the Creative Commons Attribution 4.0 International license.

**FIG 2:**
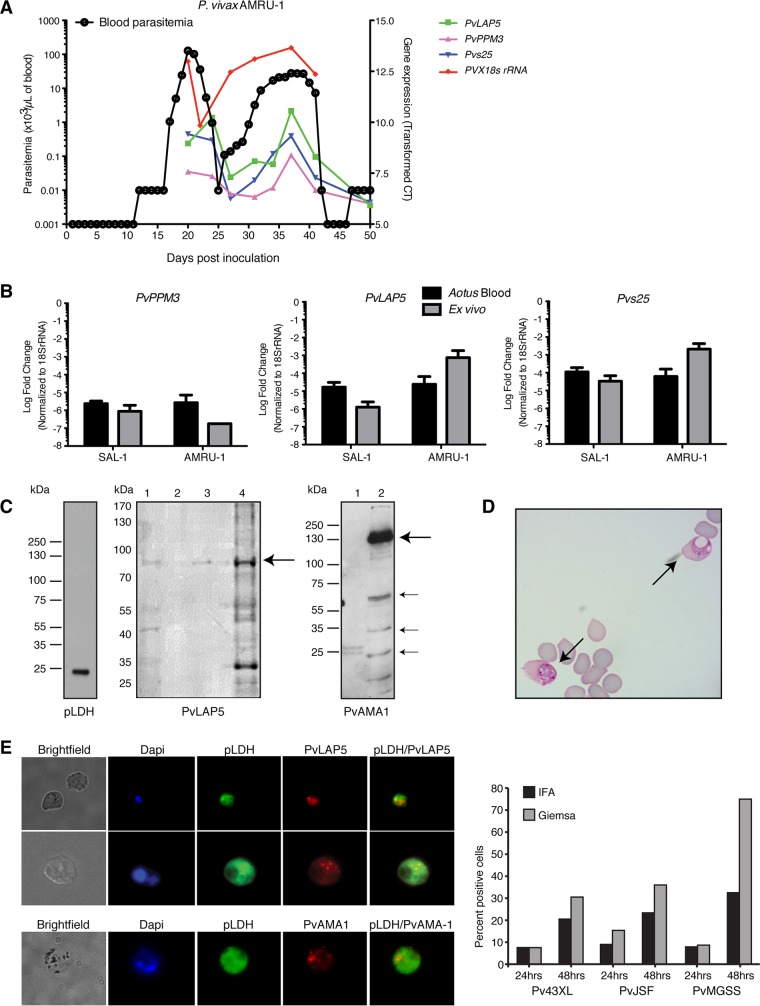
Validation of stage-specific P. vivax markers. (A) P. vivax infection dynamics in *Aotus*. Samples were collected every 3 to 5 days starting at peak parasitemia and analyzed by qRT-PCR and microscopy. qRT-PCR data were normalized to RNA input and presented as transformed cycle threshold (*C*_*T*_) values. (B) qRT-PCR quantification of candidate markers in the peripheral blood and corresponding 48-h *ex vivo* samples from *Aotus* monkeys infected with P. vivax AMRU-I or SAL-I strains (*n* = 3). Candidate markers and *Pvs25* were quantified by qRT-PCR, and values were normalized using *PVX18s rRNA*. Data are presented as transformed *C*_*T*_ values. (C) Validation of marker antibodies by Western blotting using Percoll-enriched blood samples from *Aotus* infected with P. vivax SAL-I. (Left) A single band was detected in the SAL-I *Aotus* lysate when we used mouse monoclonal pLDH antibody at a 1:5,000 dilution (approximately 1 × 10^6^ parasites/lane). (Middle) Rabbit anti-PvLAP5 peptide antibodies (1:1,000 dilution) detected full-length protein at 98.2 kDa (arrow) in lysates from infected *Aotus* (lanes 1 and 3) and the P. falciparum ortholog at 100 kDa in lysate of the P. falciparum HB3 line (lane 4, approximately 1 × 10^6^ parasites/lane from cultivated gametocytes). Lane 2, empty. (Right) Lane 1, *Aotus* lysate; lane 2, P. falciparum HB3 lysate from schizont cultures. PvAMA1 peptide antibodies (1:1,000) detected a major band and known AMA1 breakdown products in the P. falciparum HB3 schizont lysate but not in *Aotus* samples, presumably due to low yield (lane 1). (D) Image of Giemsa-stained thin blood film from *Aotus ex vivo* culture (44 h) showing a P. vivax gametocyte on the left and a vacuolated mature form on the right (arrows). (E, left) IFA images obtained with PvLAP5 (top) and PvAMA1 antibodies (bottom). (Right) Gametocyte percentages determined from PvLAP5 IFA-positive relative to total Giemsa-stained parasite counts in the *ex vivo* samples from three Brazilian P. vivax patients.

Next, qRT-PCR was used to test P. vivax-infected *Aotus* samples and *ex vivo* cultures for signals from our candidate markers. In multivariate Spearman tests, *PvPPM3* and *PvLAP5* transcript levels across *Aotus* samples and *ex vivo* cultures were both more highly correlated with *Pvs25* and each other than with *PVX18s rRNA* (Table 1), in agreement with their gametocyte-specific patterns in *ex vivo* microarrays (Fig. 1). In an AMRU-I-infected *Aotus* monkey (Fig. 2A), *PvPPM3*, *Pvs25*, and *PvLAP5* transcripts increased with parasitemia as well as *PVX18s rRNA* levels, consistent with previous reports that P. vivax gametocytemia tracks with parasitemia (3, 21). These transcription profiles of *Pvs25* and the two new gametocyte markers were also in agreement with the observed correlations of their expression (Table 1). After 48 h in *ex vivo* culture, transcripts from *PvPPM3, PvLAP5*, and *Pvs25* were detectable at levels similar to those in blood directly drawn from infected *Aotus* NHP (Fig. 2B). We did not evaluate additional markers due to the limited material obtained from the *Aotus* infections.

**TABLE 1:** Transcript correlations based on qRT-PCR analysis

Gene	Strength of correlation between gene and transcript^a^			
	PvLAP5	PVX_117730	Pvs25	PVX18s rRNA
*PvLAP5*	1	0.9042***	0.9738***	0.8024***
*PVX_117730*		1	0.9108***	0.8704***
*Pvs25*			1	0.8475***
*PVX18s rRNA*				1

a Data are the *P* values for each comparison (***, *P* < 0.0001). Data include all samples (*n* = 13) collected from *Aotus* blood and *ex vivo* time points.

### Validation of PvLAP5 gametocyte markers via immune fluorescence assays.

There is a need for antibodies that can detect marker proteins specific to P. vivax gametocytes. We therefore focused on antibodies against PvLAP5, as there is evidence for expression of its P. falciparum ortholog, PfFNPA, during gametocyte development (44, 45), and the transcription levels of *PvLAP5* and *Pvs25* are well correlated (Fig. 2A; Table 1). For these experiments, we generated rabbit antibodies against PvLAP5 peptides and tested their signals with 48-h *ex vivo* samples from *Aotus* and from human patient isolates (from Brazil). Immunoblot assays of *ex vivo Aotus* samples and uninfected controls with these antibodies demonstrated a band of the expected size (98.2 kDa) for PvLAP5 in infected blood samples (Fig. 2C). We also generated a specific antibody against the P. vivax apical membrane protein 1 (PvAMA1), the functional ortholog of the P. falciparum schizont marker and invasion determinant PfAMA1 (46) (Fig. 2C), and we employed a monoclonal antibody against parasite lactate dehydrogenase (pLDH) that detects both P. vivax and P. falciparum (47). After optimization for the immunofluorescence assay (IFA) signal, we verified specificity of the PvLAP5 and PvAMA1 antibodies on methanol-fixed *ex vivo* samples from *Aotus* and human infections. Parallel IFAs of infected red blood cells (iRBCs) *ex vivo* demonstrated that PvLAP5 stained a subset of singly nucleated parasites at the same rate as cells with morphological features of gametocytes, based on Giemsa smear (Fig. 2D and E).

### P. vivax loads and stage distributions in blood, bone marrow, liver, and lung.

**FIG 3:**
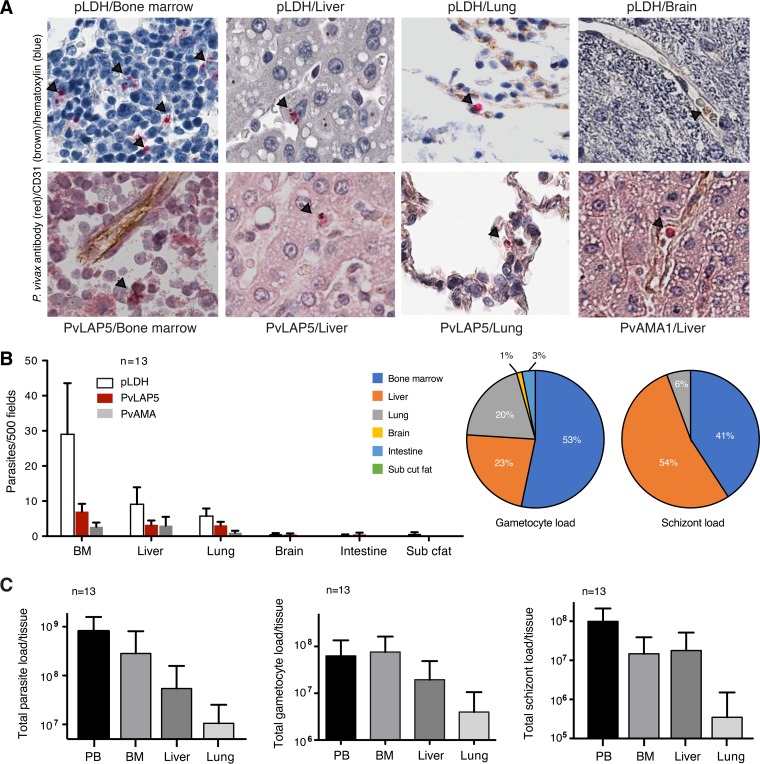
P. vivax tissue accumulation/sequestration in nonhuman primates. (A) Representative images of parasites in the immunohistochemistry (IHC) analysis of 4 tissues. pLDH, PvLAP5, and PvAMA1 antibodies were used to detect the parasite; CD31 antibodies stained the endothelium. Black arrowheads mark parasites. (B) Quantification of histological data. IHC analysis across 6 tissues from 13 monkeys was performed using parasite antibodies against pLDH, PvLAP5, and PvAMA1. Highest counts for all three antibodies were detected in bone marrow, liver, and lung. Counts represent 500 high-power fields. Values are expressed as means ± standard errors of the means (SEM). (Bottom) Pie charts showing parasite distribution across tissues based on PvLAP5 (left) and PvAMA1 counts (right). Parasites in Kupffer cells (liver) were excluded from counts. BM, bone marrow. (C) Parasite and gametocyte burden in blood and tissues. Numbers were calculated based on parasite counts from Giemsa-stained peripheral blood smears and from BM, liver, and lung tissues stained with antibodies for pLDH (all parasites) or PvLAP5 (gametocytes). (Top) Total parasite load in each tissue. (Middle) Total gametocyte load in each tissue. (Bottom) Total schizont load in each tissue. Parasites in Kupffer cells (liver) were excluded from thce ounts. Values are expressed as means ± SEM.

Considering the relative differences in stage-specific P. vivax transcripts in the human circulation (Fig. 1C), we designed experiments to directly test if P. vivax parasites might accumulate/be sequestered in vascular or extravascular niches. In a series of infections (Table S4), *Aotus* and *Saimiri* tissue samples were collected in 13 autopsies from bone marrow, lung, brain, liver, intestine, and subcutaneous fat. Histological analyses (Fig. 3) and qRT-PCR (Fig. S4) were performed similarly to those in our investigation of P. falciparum in human autopsy tissues (22), using the stage-specific markers developed in this study. Antibodies against pLDH (all parasite stages), PvAMA1 (schizonts), and PvLAP5 (gametocytes) detected parasites in the *Aotus* bone marrow, liver, and lung, while few or no parasites were detected in the brain, intestine, or subcutaneous fat (Fig. 3A and B; Table S5). Extrapolation of blood and tissue burden per animal indicated that the largest total number of parasites was present in blood circulation, while bone marrow and liver represented major tissue reservoirs for gametocytes and schizonts (Fig. 3B). We analyzed blood smear counts and total blood volume per animal to quantify the schizonts, gametocytes, and all-stage total parasites in the circulation of each animal. Parasite counts in the tissues were estimated based on corresponding counts of these forms in histological sections of defined volumes, multiplied by the estimated total tissue volume per animal (Fig. 3C). These calculations revealed average per-animal loads of approximately 8 × 10^8^ parasites in peripheral blood, 3 × 10^8^ parasites in bone marrow, and 5 × 10^7^ parasites in liver. Parasites in the bone marrow and liver thus accounted for about 30% of the total parasite burden in these animals. The data also showed that gametocytes and schizonts have enriched representation in these tissues compared to peripheral blood: gametocytes account for more than 25% of all parasites in the bone marrow and liver, compared to <10% in blood, and schizonts account for 33% in the liver compared to 12% in the blood.

10.1128/mBio.00625-18.8 TABLE S4  Details of *Aotus* and *Saimiri* infections for histological studies. Download TABLE S4, XLSX file, 0.01 MB. Copyright © 2018 Obaldia et al. 2018 Obaldia et al. This content is distributed under the terms of the Creative Commons Attribution 4.0 International license.

10.1128/mBio.00625-18.9 TABLE S5  Data from histological studies. Parasite stages were quantified from tissue sections stained with individual antibodies by immunohistochemistry. Parasites from 500 fields at ×40 magnification were counted. In tissue sections where less than 500 fields were available, the parasite counts were then extrapolated to 500 fields. Download TABLE S5, XLSX file, 0.02 MB. Copyright © 2018 Obaldia et al. 2018 Obaldia et al. This content is distributed under the terms of the Creative Commons Attribution 4.0 International license.

### P. vivax accumulation/sequestration in bone marrow parenchyma and liver sinusoids.

**FIG 4:**
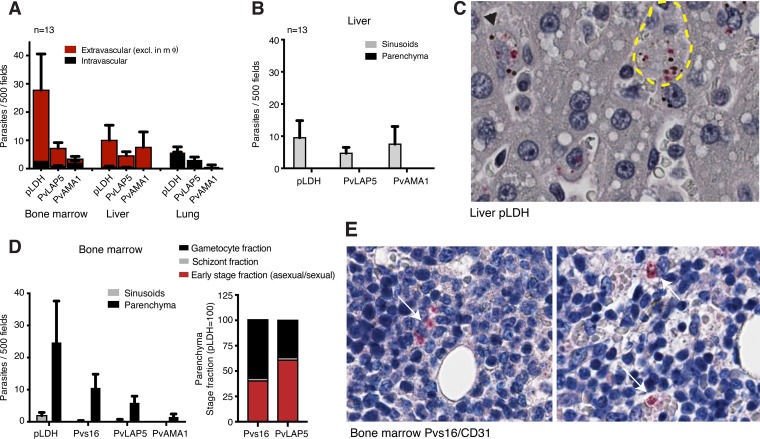
P. vivax accumulation/sequestration in bone marrow and liver. (A) Intra- and extravascular parasite distributions across tissues. Quantification of IHC data across three tissues was based on CD31 staining to differentiate intravascular from extravascular parasite localization. Parasites in macrophages were excluded from the analysis. Data demonstrated that most parasites are extravascular in the bone marrow and liver, but not in the lung. Data are from the tissues of 13 monkeys. mϕ, macrophage. (B) Extravascular parasite distribution in liver. Quantification of IHC data in liver sinusoids and parenchymas is shown. All parasites counted were present in sinusoids. Data are from the tissues of 13 monkeys. (C) Representative image of infected liver tissue. Shown are parasites in Kupffer cells (black arrowhead) and in sinusoids (yellow boundary). (D) IHC results showing extravascular parasite distribution in the bone marrow sinusoids and parenchyma. The majority of parasites counted were in the parenchyma. In bone marrow, gametocytes but not schizonts were mostly extravascular. (Right) Stage fraction in BM parenchyma (stacked bar with pLDH [taken as 100]). Schizonts were quantified based on PvAMA1-positive parasites, and gametocytes were quantified based on Pvs16- or PvLAP5-positive parasites. The remainder (after subtracting PvAMA1 and Pvs16/PvLAP5 counts from pLDH counts) were cataloged as the early-stage fraction. Data are from the tissues of 13 monkeys. (E) Representative images of infected bone marrow tissue. Extravascular parasites are marked with white arrows; the image on the right shows two parasites associated with erythroblastic islands.

Microscopic studies of the tissue specimens further demonstrated that the vast majority of parasites in infected bone marrow and liver tissue were located outside of the vasculature, in contrast to parasites in lung tissue, which were detectable only in the vasculature (Fig. 4A; Table S5). Excluding parasites cleared by Kupffer cells, extravascular parasites were observed in sinusoids but not in the hematopoietic region of the parenchyma (Fig. 4B and C). In the bone marrow, the majority of parasites, including schizonts and gametocytes, localized to the parenchyma, while only a small fraction was detected in sinusoids (Fig. 4D and E). In the bone marrow, the number of parasites detected by pLDH was far greater than with PvLAP5 and PvAMA1, suggesting that the majority of parasites were early stages not detected by either of these 2 antibodies. Indeed, specific gametocyte staining with antibodies against the pan-gametocyte marker Pvs16 (48) marked about half of all pLDH^+^ cells in the bone marrow parenchyma. A similar observation was made when we stained infected red blood cells with the P. falciparum ortholog Pfs16 in human bone marrow (22).

## DISCUSSION

Observations of moderate numbers of mature as well as immature P. vivax stages in the circulation have probably contributed to general misconceptions that P. vivax malaria is benign, although evidence has repeatedly shown that this is a disease with tremendous morbidity and mortality (4, 9, 49–51). Parasite accumulation/sequestration in tissues is thought be an important contributor to the pathogenesis of severe vivax malaria (49). Human case studies (25, 51, 52) have demonstrated P. vivax stages in spleen, lung, and bone marrow, analogous to findings of accumulation/sequestration in infections with other species of *Plasmodium* (53–57). P. vivax is also found in the deep tissues of NHP (58, 59), and the presence of a significant tissue reservoir of P. vivax and related parasites, such as *Plasmodium cynomolgi*, has been predicted based on measured blood parasitemia compared to observed parasite growth dynamics in infections of humans and animals (60).

In the present study, we used specific antibodies and quantitative histological analysis to develop systematic analysis of P. vivax stage distributions in human blood samples and major tissues of the *Aotus* and *Saimiri* NHP models. Comparative analysis of transcriptional profiles from P. vivax *ex vivo* and P. falciparum *in vitro* time courses indicates similar dynamics of asexual and sexual gene expression, even though the development time for P. vivax gametocytes is much shorter than that for P. falciparum gametocytes. These similarities demonstrate conservation of essential pathways of development despite deep evolutionary divisions between the distinct species.

Our results indicate relatively lower counts of mature asexual and immature gametocytes in the circulating blood of P. vivax patients. These findings are consistent with PvAMA1 and PvLAP5 staining results from the tissues of infected *Aotus* and *Saimiri* monkeys. The histological data demonstrate a major fraction of gametocytes in the parenchyma of the bone marrow, whereas asexual schizont forms are enriched to a somewhat lesser extent in this region of the bone marrow as well as in sinusoids of the liver. Smaller fractions of gametocytes and schizonts are represented in the blood circulation. These findings corroborate the hypothesis that subpopulations of asexual and gametocyte stages accumulate/sequester from the circulating blood during P. vivax infection, similar to our previous observations with P. falciparum. Use of additional stage-specific markers and accumulation/sequestration studies in spleen-intact NHP as well as human autopsies will be needed for further information on the importance of these stage distributions for P. vivax transmission and pathogenesis.

Although recent data suggest that rosetting may contribute to P. vivax vascular accumulation/sequestration (61, 62), the host-parasite interactions underlying the distribution patterns observed in our study are unknown. A significant fraction of parasites in the bone marrow parenchyma (>40%) are young stages (positive for pLDH but negative for PvLAP5, Pvs16, and PvAMA1), confirming detection of significant levels of ring-stage parasites in bone marrow aspirates (25). P. vivax invades young reticulocytes that are prevalent in the bone marrow parenchyma (24), so that early stages of P. vivax development may have a preference for this hematopoietic environment (63). This scenario may have implications for efforts to establish P. vivax culture *in vitro*, as bone marrow reticulocytes are phenotypically different from the more mature forms present in the blood circulation (64). Localization of the parasites to a reservoir in the bone marrow may help to explain why many P. vivax infections in Africa have gone undetected (5), and it also raises the possibility that recurrent blood-stage parasitemias can arise from bone marrow as well as from relapses of hypnozoite stages in the liver.

## MATERIALS AND METHODS

### P. vivax gene annotation through ortholog mapping.

We mapped P. vivax transcripts by using the stage and cluster annotations of P. falciparum (29) after identification of their orthologs in PlasmoDB (http://www.plasmodb.org). Only syntenic ortholog mappings were considered. For asexual annotation, 3,990 out of 4,396 asexual P. falciparum genes had P. vivax orthologs. Most variant genes were excluded due to lack of syntenic orthologs. P. vivax genes were then labeled for cluster and stage according to their P. falciparum gene ortholog; 244 out of 249 asexual clusters had at least one corresponding P. vivax gene. Out of the 591gametocyte genes annotated as gametocyte specific in a previous report (29), 527 had corresponding P. vivax orthologs. All 29 gametocyte clusters had at least one P. vivax gene representative, and 27 clusters had at least five genes. These annotations were used for stage-specific P. vivax gene expression analysis.

### Microarray data analysis.

We obtained time course and cross-sectional microarray P. vivax expression profiles of infected blood samples from references 31 and 34, respectively. The expression values from reference 31 were already filtered, normalized, and transformed. Expression values from reference 34 were also normalized and only log-transformed and averaged across technical replicates. For comparison of gene expression levels of different stages (Fig. 1C, box plots), expression levels were averaged across all genes with the same stage annotation. *P* values were assigned based on two-sample paired *t* tests. Similarly, gene expression levels were averaged clusterwise for comparison of different clusters (Fig. 1C, heat maps).

### Animals, infection protocol, and sample collection for bloodstream P. vivax analysis. (i) Animals.

Fourteen spleen-intact male and female laboratory-bred Aotus lemurinus lemurinus monkeys karyotypes VIII and IX (65), with body weights between 758 and 829 g, were used as donors, experimental subjects, or controls. The animals were housed at Gorgas Memorial Institute of Health Studies (ICGES) in Panama and cared and maintained as described elsewhere (38). All animals received daily veterinary care, which included regular physical examinations and body weight determinations at least four times a year, deworming (mebendazol, Pantelmin; Janssen-Cilag, New Brunswick, NJ), as well as vitamin B_12 _(Catosal; Bayer, Shawnee Mission, KS) and vitamins A, D, and E (Vigantol; Bayer) injections. Rooms had climate control with 12 air changes per hour, min-max temperatures set to 70 to 76°F and 70 to 80% humidity, with a fluorescent red/white light 12-h cycle starting at 03:00 p.m. A balanced mixture of fruits and laboratory primate chow was provided to meet their caloric requirements. Water was administered daily in plastic bottles fitted with a zip tube (Girton; Millville, PA). The animals were kept in pairs in stainless steel 4-unit quad cages (Lab Products Inc., Seaford, DE) with dimensions of 27 by 23.5 by 29.5 inches. Each cage was fitted with a 3/4-inch-diameter polyvinyl chloride (PVC) pipe perch placed across 2/3 of the length of the cage and a 6-inch-diameter, 14.5-inch-long PVC T pipe nest box attached to the roof and back of the cage with cable zip ties. Cages were routinely cleaned and sterilized at 180°F at weekly intervals in a cage washing machine (Steris, Erie, PA). During experimental infections, the animals did not receive analgesics, nor were they trained for special procedures. The experimental protocol was approved by the Gorgas Memorial Institute of Health Studies, Institutional Laboratory Animal Care and Use Committee (CIUCAL) in accordance with procedures described in the *Guide for the Care and Use of Laboratory Animals* (76) (protocol approval number 2011/02). All experiments described here were performed in accordance with the approved guidelines.

### (ii) Infection protocol.

A frozen parasite stabilate of P. vivax SAL-I or AMRU-I was thawed and inoculated into the saphenous vein of experimental spleen-intact *Aotus* monkeys as described previously (66). Giemsa-stained thick blood smears were then examined daily from a prick in the marginal ear vein, starting on day 5 postinoculation (p.i.), and parasitemia was evaluated and reported as the number of parasites per microliter using the method described in 1932 by Earle and Perez (67).

### (iii) Sample collection.

For expansion of P. vivax parasite populations, we inoculated one donor animal each with AMRU-I and SAL-I, respectively, and passaged the parasites in four experimental animals once parasitemia reached 5 × 10^3^/μl (Table S2). Parasitemia was monitored, and the animals were bled once for experimental samples (Fig. S2); when parasitemia reached a peak of ≥8.0 × 10^3^ parasites per µliter (between days 12 and 15 postinfection), 3.5 ml of blood was collected from the femoral vein in sodium citrate anticoagulant and spun down, and the plasma was separated and frozen for later use. The iRBC pellet was washed three times with phosphate-buffered saline (PBS; pH 7.2) and further divided: one aliquot containing 500 µl of packed RBCs was preserved in Glycerolyte and stored in liquid nitrogen as a stock repository; the remaining 1.5 ml of packed RBCs was split into 4 aliquots for subsequent use in qRT-PCR, Western blot analysis, IFA, and *ex vivo* culture. For qRT-PCR, packed RBCs were resuspended in 2.5 ml of McCoy’s medium supplemented with 20% human serum and passed once through a CF11 cellulose column previously stabilized with PBS (pH 7.2) to exclude white blood cells from the sample (68). The recovered RBC eluate was further centrifuged, washed three more times in PBS, and adjusted to a 20% hematocrit with McCoy’s medium to a suspension volume of ~1.3 ml. At this point, blood was mixed with 5 parts of Trizol for mRNA preservation. For Western blot analysis, packed RBCs were treated with saponin at 0.15% and centrifuged, and the pellet was washed, resuspended in SDS sample buffer, and frozen until further use. For IFA, the RBC sample was diluted 1:1,000 in PBS, spotted onto slides, fixed with methanol, and frozen until used for either IFA or Giemsa staining. For *ex vivo* culture, RBCs were overlaid onto a 45% Percoll cushion as described previously (69–71) and then centrifuged, and the band containing enriched mature P. vivax-infected RBCs (trophozoites, schizonts, and gametocytes) was collected and processed for Western blotting and IFA smears as described above. The pellet containing mostly ring forms was washed in PBS, resuspended in 5 ml of McCoys’s complete medium, and cultured in Corning Costar tissue culture 6-well plates (Sigma-Aldrich, St. Louis, MO) at 37°C in a culture hermetic bubble in an atmosphere of 90% N_2_, 5% CO_2_, and 5% O_2_ for 48 to 96 h, replacing the gas mixture every 24 h without changing the medium. Giemsa-stained smears were taken at 24 and 48 h, and at 48 h additional aliquots were collected for qRT-PCR and IFA experiments as described above. Finally, the animals were rescued by treatment with mefloquine (MQ) at 20 mg/kg of body weight orally once to end the experiment.

To measure the dynamics of infection and gametocytogenesis, we treated an animal with CQ. This approach is based on the observation that gametocytogenesis is enhanced when subjects are treated with CQ or pyrimethamine, as it has been demonstrated in the Plasmodium chabaudi rodent malaria model (72–75). Specifically, we used the CQ-resistant P. vivax AMRU-I strain for this experiment, assuming that CQ treatment would sufficiently stress the parasite population to increase gametocyte production without clearing the asexual infection. We infected one *Aotus* monkey with P. vivax AMRU-I, and the animal was then treated at peak parasitemia and at recrudescence with CQ at 15 mg/kg orally for 3 days. During infection follow-up for the last 50 days p.i., blood samples were collected at 9 time points, approximately 3 to 5 days apart, to measure dynamics of infection by qRT-PCR and Giemsa smear.

### Nonhuman primate terminal studies.

All NHP animal acquisition, transportation, housing, care, procedures, and release from studies were performed according to the National Institutes of Health (NIH) Animal Research Advisory Committee (NIH ARAC) guidelines, protocols approved by the National Institute of Allergy and Infectious Diseases (NIAID) Animal Care and Use Committee (NIAID ACUC), and in compliance with the Animal Welfare Act and the *Guide for the Care and Use of Laboratory Animals* (76). Briefly, *Saimiri* and *Aotus* monkeys were socially housed, consistent with the experimental design, in accord with the NIAID DIR Animal Care and Use Program Policy on Social Housing of Nonhuman Primates. All husbandry was consistent with the current state of the art for New World nonhuman primate care, including maximum quiet, fully enclosed nesting areas, a 12-h light-dark cycle, and safe toys. Daily cleaning and technician health checks were conducted as quietly and unobtrusively as possible. Individual animal records were taken to reflect all procedures conducted. A balanced mixture of fruits and laboratory primate chow were provided to meet their caloric requirements.

Only splenectomized animals that participated in multiple malaria studies and were eligible for final bleed were utilized for postmortem sample collection by NIH pathologists.

Approximately 10^3^ to 10^5^ P. vivax pRBCs from cryopreserved or fresh NHP blood were utilized to infect animals intravenously (i.v.). Animals were monitored weekly or daily based on blood smears, hematocrit, and weight. Animals in this study experienced only subclinical signs from the infection (namely, blood RBC parasitemia and minor hematocrit depression). Any monkey that reached a parasitemia of 5% or more or a hematocrit of 25% or less was immediately treated for cure of parasites, including on weekends and holidays, with MQ (25 mg, single dose, orally) or Malarone (weight-adjusted pediatric dose of 25 mg/kg/day atovaquone plus 10 mg/kg/day Proguanil, orally for 3 days). Criteria for consideration of euthanasia (at the discretion of the attending veterinarian, after consultation with the Principal Investigator) included the initial and current hematocrit, initial and current degree of parasitemia, other indicators of response to the antimalarial drug treatment, and the general health condition of the animal. No animal suffered unnecessarily. Euthanasia was performed in accordance with the *Guide for the Care and Use of Laboratory Animals* (76).

### P. vivax *ex vivo* culture from human patient samples.

Cryopreserved Brazilian P. vivax isolates were obtained through the “Efficacy of Chloroquine (CQ) Alone Compared to Concomitant CQ and Primaquine for P. vivax Infection” clinical trial (NCT02691910) with written informed consent from all patients (Institutional Review Board of the Institute of Biomedical Sciences, University of São Paulo, Brazil [1169/CEPSH, 2014]). Cryopreserved isolates were thawed as previously described (77). Subsequently, P. vivax ring-stage parasites were enriched on a 1.080 g/ml KCl-Percoll gradient. Briefly, 1.080 g/ml KCl-Percoll was achieved by combining KCl high isotonic Percoll (10 mM HEPES, 115 mM KCl, 12 mM NaCl, final) at 35.97% with the KCl high buffer at 64.03%, as described by Roobsoong et al. (78). Density was confirmed with a DMA 35 portable density meter (Anton Paar; Graz, Austria). Density gradients were assembled by layering one part thawed cells in incomplete Iscove’s modified Dulbecco’s medium (IMDM) (3 to 50% hematocrit) on one part density gradient and then centrifuged for 15 min at 1,200 × *g*. Enriched parasites were collected from the interface, washed twice with incomplete IMDM, and finally resuspended at 1 to 2% hematocrit in completed IMDM (10% heat-inactivated pooled AB^+^ human sera from Interstate Blood Bank, Inc. [Memphis, TN] and 50 mg/ml gentamicin) and moved into culture. Cultures were maintained at 37°C in 5% CO_2_, 1% O_2_, and N_2_ to balance. Parasitemia and asexual and gametocyte maturation were assessed by either light microscopy analysis or methanol-fixed and Hemacolor-stained cytospins (Shandon Southern Instruments, Sewickley, PA).

### Sample processing and RNA extraction.

For blood samples (*Aotus*, *ex vivo* samples), RNA was extracted from Trizol samples by using a Qiagen RNeasy Plus kit including a gDNA eliminator column (Qiagen, Valencia, CA), according to the manufacturer’s protocols.

Tissue samples from monkeys were either fresh frozen or stored in RNAlater (Qiagen) for subsequent RNA analyses. All tissues were completely homogenized in RLT lysis buffer (Qiagen) and using a Polytron homogenizer (Kinematica). Homogenized tissue lysate was centrifuged at 15,000 rpm for 15 min to remove undigested material (pellet), and the resulting clear supernatant was subsequently used for RNA extraction using an RNeasy plus minikit (Qiagen). The lysate was passed through gDNA eliminator columns (Qiagen) to remove genomic DNA, and RNA was extracted according to the manufacturer’s protocol. RNA was eluted in nuclease-free water. An additional gDNA removal step was performed using Turbo DNase (Ambion) treatment for subsequent qRT-PCR analyses. RNA concentrations were measured in a NanoDrop ND-1000 spectrophotometer (Thermo Fisher Scientific, Cambridge, MA),and samples were stored −80°C until cDNA synthesis.

### cDNA synthesis.

Reverse transcription was performed with an Invitrogen SuperScript III first-strand system for RT-PCR (Life Technologies, Inc., Carlsbad, CA). Briefly, 8 µl containing ~1 pg to 5 µg of total RNA was extracted by the Trizol method described above and used as the template in a 20-µl volume. After the reaction was stopped, RNase H was added for 20 min and then the samples were stored at −20°C until use. One extra reaction mixture without reverse transcriptase (RT-) was carried out as a negative control for genomic DNA contamination in all samples.

### Primers for the qRT-PCR assay.

To quantify stage-specific gene expression of selected P. vivax markers by qRT-PCR, we designed forward and reverse primers by using the following criteria: (i) maximum size of 20 bp; (ii) GC content of 55%; (iii) melting temperature (*T*_*m*_) of 60°C; (iv) PCR product length of 70 to 200 bp. In addition, primers for the candidate gametocyte markers and the constitutive marker *PVX_091645* were designed such that one of the two primers spanned an exon-exon junction, in order to minimize amplification from gDNA. Primers were synthesized by GenScript (Piscataway, NJ) and optimized on synthetic cDNA templates as described in Results and for Fig. 2A. For standard controls, we used *Pvs25* (*PVX_111175*) and *Pv18s rRNA*.

PCR amplification was performed in 20-µl volumes with a Fast SYBR Green qPCR master mix system from Life Technologies, Inc., and an Applied Biosystems ViiA 7 real-time qPCR thermocycler system.

### Generation of peptide antibodies against PvLAP5 and PvAMA1.

Peptides used for rabbit immunizations are listed in Table S3. Antisera were generated by immunization of one rabbit per construct using standard protocols (GenScript, Piscataway, NJ). Preimmune serum samples were harvested for each study animal on the day prior to immunization (day −1) to immunization. On day zero, each animal was immunized by intraperitoneal injection (i.p.) with a primary dose of 50 µg of recombinant protein in Freund’s complete adjuvant. This was followed by three boosts of 25 µg protein in Freund’s incomplete adjuvant administered i.p. on days 14, 25, and 56. Antisera were collected on day 63 and tested in an enzyme-linked immunosorbent assay for reactivity against the recombinant protein relative to preimmune control serum samples.

### Western blot analysis.

For immunoblot analysis, 375 µl of peripheral blood at a peak parasitemia of ~8.0 × 10^3^ parasites/µl was collected from *Aotus* monkeys infected with P. vivax SAL-I; the blood samples were treated with 0.1% saponin in 1× PBS buffer to release parasites. Saponin-lysed parasites (3.0 × 10^6^) were resuspended in 10 volumes (15 µl) of SDS lysis buffer, and 5 µl (1.0 × 10^6^) of each sample was used for Western blotting. Blood from an uninfected *Aotus* monkey was used as a negative control. Mouse anti-pLDH antibodies at a 1:5,000 dilution, rabbit anti-PvLAP5 antibodies (GenScript) at a 1:1,000 dilution, and rabbit anti-PvAMA1 antibodies (GenScript) at a 1:1,000 dilution were used. Horseradish peroxidase (HRP)-conjugated goat anti-mouse IgG (H+L) and goat anti-rabbit IgG (H+L) secondary antibodies were used for detection.

### Immunofluorescence assays.

P. vivax AMRU-I and SAL-I samples from each inoculated *Aotus* animal were collected for thick and thin blood smear examination by Giemsa staining and bright-field microscopy. *Aotus* blood and *ex vivo* samples were spotted onto 10-well slides, fixed in methanol, and snap-frozen until further use. For IFA, slides were thawed and processed for staining. For antibody optimization, slides were incubated in a humid chamber for 1 h with serial dilutions of 1:500 to 1:5,000 antibody titers in bovine serum albumin-PBS (pH 7.2) buffer and then washed thoroughly with PBS 3 times. Samples were further labeled with secondary goat anti-rabbit Alexa Fluor 488-fluorescein isothiocyanate–conjugated antibodies (Invitrogen Molecular Probes, Eugene, OR), diluted 1:500, and incubated for 30 min. After a final wash with PBS, samples were mounted and counterstained with Vectashield 4′,6-diamidino-2-phenylindole (Vector Laboratories, Burlingame, CA) and analyzed with a Nikon Eclipse TE300 inverted fluorescence microscope.

### Histological assays.

Formalin-fixed tissues from monkeys were embedded in paraffin blocks, and sections of 5-µm thickness were obtained for each tissue. For immunohistochemistry analysis, sections were processed through deparaffinization steps in xylene followed by dehydration steps through a graded ethanol series (100% to 50%). Antigen retrieval was performed by incubating slides at 95°C in a steamer for 30 min and using a universal antigen retrieval reagent (R&D Systems). Following antigen retrieval, blocking was performed using Tris-buffered saline blocking buffer (Thermo Scientific) for 20 min, followed by 10 min each with avidin and biotin blocking buffers (Invitrogen) to block endogenous biotin and avidin, respectively. Double labeling of parasite-specific antigens (pLDH, PvLAP5, PvAMA1, and Pvs16) and blood vessels (CD31) was performed by the following protocol of antibody dilutions and incubations. Mouse monoclonal anti-pLDH antibodies were used at 1:1,000 dilution in combination with rabbit anti-CD31 antibodies (Abcam, Inc.) at 1:20 dilution. Rabbit anti-PvLAP5 antibodies (1:500), rabbit anti-PvAMA1 antibodies (1:500), or rat anti-Pvs16 antibodies (1:500) were each used in combination (1:20) with mouse anti-CD31 antibodies (Abcam, Inc.). Primary antibodies were diluted in blocking buffer and incubated overnight at 4°C. Secondary antibodies were selected to label parasites in red and blood vessels in brown. HRP-conjugated goat anti-rabbit or anti-rat–HRP antibodies and a biotin conjugate of the F(ab')2 fragment of goat anti-mouse IgG (H+L) antibodies were used (Invitrogen) (diluted to 1:500 in universal blocking buffer), followed by streptavidin conjugated to alkaline phosphatase (AP; Thermo Scientific) (diluted 1:3,000 in universal blocking buffer). For the development of signal, 3,3′-diaminobenzidine (DAB) chromogen reagent (Thermo Scientific) was added for 20 min for development of brown color. The slides were washed in water, and Fast Red 4 TR/naphthol AS-MX substrate reagent (Sigma-Aldrich) was applied for 5 min. Slides were subsequently rinsed in water and counterstained in Mayer’s hematoxylin and mounted in aqueous mounting medium. Images of the slides were captured using a whole-slide imaging system, and parasites were quantified by counting 500 consecutive high-power fields at 400× magnification. Intravascular and extravascular parasite localization was quantified with respect to CD31-positive blood vessels.

### Calculation of tissue and blood parasite burdens. (i) Bone marrow.

For calculation of parasite burdens, first the volume of the bone marrow from each animal was calculated using total body weight and the assumption it represented 4% of body weight and had a density of 1.03 g/cm^3^ (79). Second the volume of the slide measurement (500 high-power fields [hpf]) was calculated as follows: length (160 µm) × width (160 µm) × section depth (5 µm), i.e., 3.2 × 10^−6^ cm^3^. The volume of bone marrow was then divided by the volume of the slide to give the number of bone marrow units per animal. The resulting number was multiplied by the number of parasites per 500 hpf to give the total number of parasites in bone marrow for each animal.

### (ii) Liver.

For liver burdens, the volume of the liver was calculated using total body weight and an assumption it represented 27 ml per kg (79). The volume of the liver was then divided by the volume of the slide (see above, 3.2 × 10^−6^ cm^3^) to give the number of liver units per animal. The resulting number was multiplied by the number of parasites per 500 hpf to give the total number of parasites in liver for each animal.

### (iii) Lung.

For lung burdens, the volume of the lung was calculated using total body weight and an assumption it represented 7.7 ml per kg (79). The volume of the lung was then divided by the volume of the slide (see above, 3.2 × 10^−6^ cm^3^) to give the number of lung units per animal. The resulting number was multiplied by the number of parasites per 500 hpf to give the total number of parasites in lungs for each animal.

### (iv) Peripheral blood.

For peripheral blood parasite burdens, the total volume of blood was calculated using total body weight and an assumption it represented 50 ml per kg (79); based on that volume, the total RBC count was calculated using an estimate of 5 × 10^6^ red cells/µl. This number was multiplied by the measured parasitemia to give the total number of parasites in peripheral blood for each animal.
