# Supplementary figures and images for: Bone Marrow Is a Major Parasite Reservoir in Plasmodium vivax Infection
Source: mBio. 2018 May 8;9(3):e00625-18. doi: 10.1128/mBio.00625-18 (PMC5941073; doi:10.1128/mBio.00625-18)

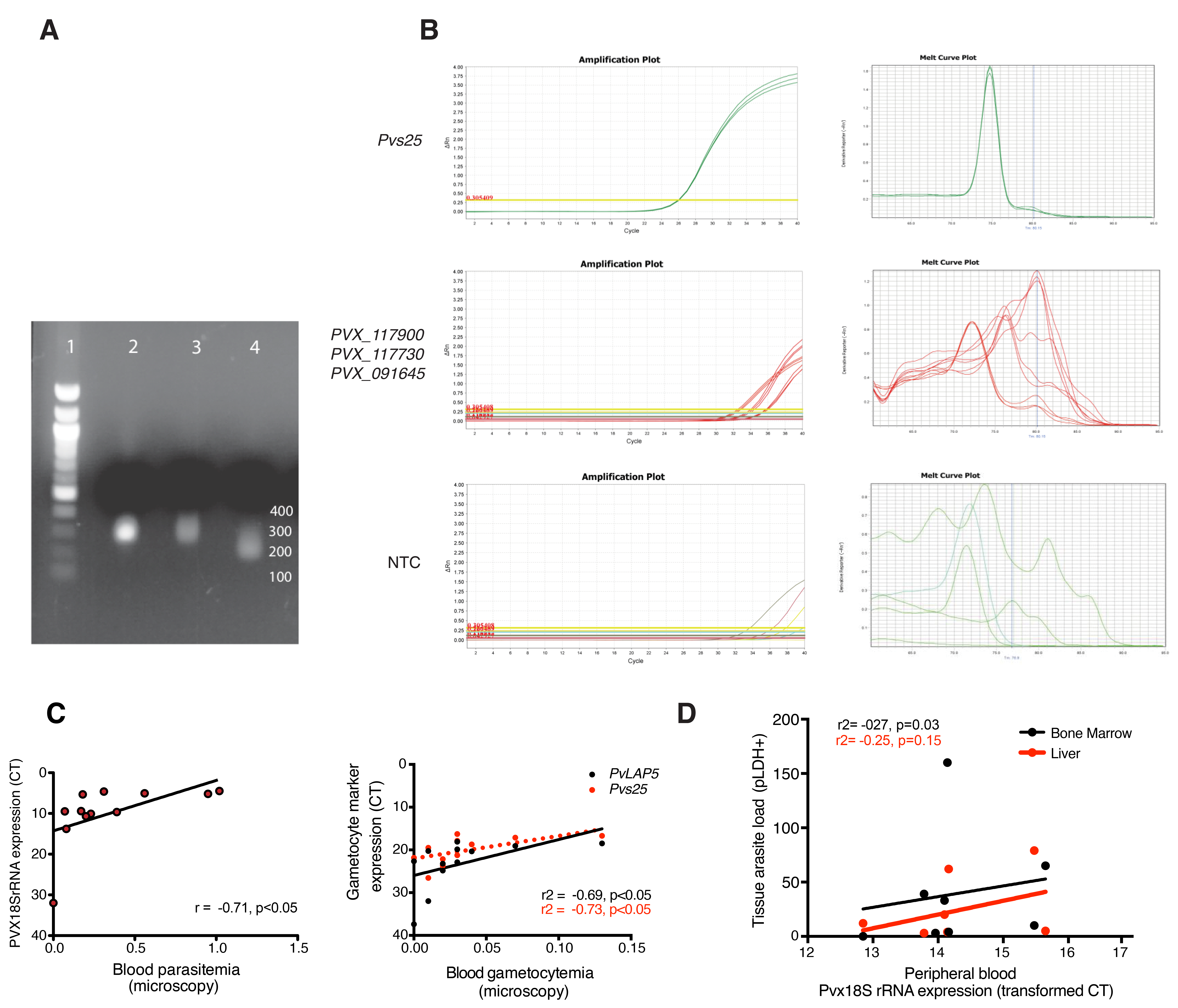

Supplement: FIG S4 [file mbo003183873sf4.tif]
